# Adsorption of allergen protein at surfaces

**DOI:** 10.1186/2045-7022-1-S1-P10

**Published:** 2011-08-12

**Authors:** Nasser Al Shabib

**Affiliations:** 1 Leeds University, Food science and Nutrition, Leeds, UK

## 

Cleaning of processing equipment in the food industry and of surfaces in catering and domestic environments is a key issue in prevention of accidental exposure of individuals with food allergy to allergenic foods. Despite this, little systematic work has been carried out understanding adsorption processes and the effectiveness or otherwise of cleaning procedures. The limiting factor in studying allergenic proteins at surfaces is our inability to reliably detect and quantify allergen proteins that may have undergone denaturation, whether through the adsorption process or through other factors such as thermal treatment. Antibody-based methods are notoriously susceptible to changed responses to different forms of the same protein - despite the commercial availability of diagnostic kits for testing 'swabbed' surfaces. We have tried to use a specific ELISA-based assay for ovomucoid alongside a non-specific chemical method for protein detection to study adsorption of the protein to different surfaces (stainless steel, formica and glass). Protein recovery was effected with the use of cotton swabs. Statistical analysis of the data (ANOVA) was carried out to determine the effect of different factors on the efficiency of 'sticking' of the protein at the surfaces. The results (p< 0.05) showed significant effects from some factors but not all. We were also able to compare the effectiveness of the chemical and ELISA methods. This is the first time that a validated immunochemical method and a chemical assay have been used to investigate the behaviour of allergen proteins at surfaces and forms part of a comprehensive study on the behaviour of allergen proteins on different surfaces.

